# Solvometallurgy as Alternative to Pyro- and Hydrometallurgy for Lithium, Cobalt, Nickel, and Manganese Extraction from Black Mass Processing: State of the Art

**DOI:** 10.3390/ma18122761

**Published:** 2025-06-12

**Authors:** Alessandra Zanoletti, Alberto Mannu, Antonella Cornelio

**Affiliations:** INSTM and Chemistry for Technologies Laboratory, Department of Mechanical and Industrial Engineering, University of Brescia, Via Branze 38, 25123 Brescia, Italy; alessandra.zanoletti@unibs.it (A.Z.); antonella.cornelio@unibs.it (A.C.)

**Keywords:** solvometallurgy, black mass recycling, low-melting mixtures solvents (LoMMSs), critical metals recovery, lithium-ion battery waste

## Abstract

The rapid growth in lithium-ion battery (LIB) demand has underscored the urgent need for sustainable recycling methods to recover critical metals such as lithium, cobalt, nickel, and manganese. Traditional pyrometallurgical and hydrometallurgical approaches often suffer from high energy consumption, environmental impact, and limited metal selectivity. As an emerging alternative, solvometallurgy, and in particular the use of low-melting mixtures solvents, including deep eutectic solvents, offers a low-temperature, tunable, and potentially more environmentally compatible pathway for black mass processing. This review presents a comprehensive assessment of the recent advances (2020–2025) in the application of LoMMSs for metal recovery from LCO and NCM cathodes, analyzing 71 reported systems across binary, ternary, hydrated, and non-ChCl-based solvent families. Extraction efficiencies, reaction kinetics, coordination mechanisms, and solvent recyclability are critically evaluated, highlighting how solvent structure influences performance and selectivity. Particular attention is given to the challenges of lithium recovery, solvent degradation, and environmental trade-offs such as energy usage, waste generation, and chemical stability. A comparative synthesis identifies the most promising systems based on their mechanistic behavior and industrial relevance. The future outlook emphasizes the need for greener formulations, enhanced lithium selectivity, and life-cycle integration to support circular economy goals in battery recycling.

## 1. Introduction

The global shift toward electrification, particularly in transport and renewable energy, is driving strong growth in lithium-ion battery (LIB) deployment. The LIB market, valued at over $60 billion in 2023, is projected to surpass $185 billion by 2030, with electric vehicles (EVs) expected to account for over 80% of this demand [1]. Parallel to this expansion is a sharp rise in end-of-life (EOL) battery volumes, forecast to reach 11 million metric tons annually by 2030, potentially exceeding 25 million tons by 2040 [2]. This scenario raises two pressing challenges: the safe disposal of hazardous spent batteries and the recovery of strategic elements such as lithium, cobalt, nickel, and manganese. These materials are geographically concentrated; over 70% of cobalt originates from the Democratic Republic of the Congo, and China controls more than 60% of lithium refining capacity [3]. As global demand for lithium is expected to quadruple by 2035, and for cobalt and nickel it is expected to double, concerns over supply chain stability and geopolitical dependencies have intensified. In response, the European Union (EU) has strengthened battery waste regulations, classifying the black mass (BM) produced by spent LIBs as hazardous waste [4] to ensure its domestic processing. In fact, it typically contains heavy metals (Co, Ni, Mn, Cu), residual electrolytes, and fluorinated binders (e.g., PVDF), many of which are toxic, flammable, or reactive. Exposure to air or moisture can trigger thermal runaway, HF release, or metal dust dispersion, posing significant health and environmental hazards. The updated classification prohibits shipment to non-OECD countries and mandates handling under strict Basel Convention and Waste Shipment Regulation (WSR) guidelines, aligning with the EU’s circular economy and strategic autonomy goals [5]. BM, rich in critical metals, represents a key secondary resource and is central to the EU Battery Regulation’s goals of circularity and strategic autonomy.

The recovery of strategic elements becomes critically important to reduce the need for their importation from other countries. Indeed, their occurrence is highly concentrated in specific regions of the world and faces several issues related to their supply chain. Recent studies emphasize that critical raw material supply chains, particularly for lithium, are not only geographically concentrated but also structurally fragile due to opaque patterns of ownership and control [6]. While countries like Australia, Chile, and China dominate lithium extraction and refining, control over these operations is increasingly exercised by a limited number of transnational firms, many of which are influenced or owned by state actors such as China. This complexity in governance creates strategic vulnerabilities, as over 33% of global lithium production is estimated to be controlled by Chinese entities through direct or indirect ownership, despite being geographically dispersed. The U.S.–China technological rivalry, compounded by export restrictions, investment screening, and potential “lithium cartels”, underscores the geopolitical dimensions of supply chain resilience, prompting governments to reconsider industrial policy, foreign investment rules, and resource sovereignty frameworks.

The current complex geopolitical landscape, further exacerbated by conflicts over the control of mineral deposits, calls for the development of technologies aimed at their recovery, thereby also mitigating the significant environmental impact often associated with primary extraction. To enable the recovery of strategic metals, a series of pretreatment steps must be carried out on end-of-life batteries, including discharge, dismantling, magnetic separation, and shredding, with the aim of obtaining the BM, a fine powder rich in graphite and complex metal oxides. The composition of metal oxides in the black mass depends on the type of battery used as feedstock. Different cathode chemistries are tailored to specific applications; among the most common are lithium cobalt oxide (LCO), composed of lithium and cobalt oxides, and lithium nickel manganese cobalt oxide (NCM), which contains lithium, nickel, manganese, and cobalt oxides in varying ratios [7]. LCO-type BM typically originates from small electronic devices such as smartphones, whereas NCM-type cathodes are predominantly used in electric vehicles [8]. It is important to highlight that the chemical composition of the cathode materials directly influences battery performance and lifespan. Regardless of the specific chemistry, a key objective in recycling is the efficient recovery of the strategic metals they contain, due to their high economic value and criticality for various technological applications [9]. Moreover, on 5 March 2025, the EU updated the European Waste Catalogue, officially classifying BM as hazardous waste [10]. This regulatory change is intended to strengthen the control of BM shipments and ensure that this critical material remains within the European economy. By doing so, it supports the objectives of the EU Battery Regulation [11], which promotes a circular economy and aims to enhance the Union’s strategic autonomy. This highlights that the BM recycling market is expected to expand significantly in the coming years, driven by the rapid growth of battery production.

Over the years, several types of processes have been developed to recover strategic metals contained in BM, with the earliest approaches being pyrometallurgical and hydrometallurgical methods. Pyrometallurgical processes typically occur at very high temperatures, often exceeding 1400 °C, and are widely employed due to their compatibility with various battery chemistries, operational flexibility, and ease of scale-up [11]. Graphite, which is present in the BM, plays a key role in the process, as it partially contributes to the reduction in metal oxides and partially combusts to form CO_2_ [12,13]. At the end of the process, metals are recovered in the form of a metal alloy. Although this approach enables high recovery rates of cobalt and nickel, it results in partial losses of lithium and manganese. They primarily remain concentrated in the slag, making their recovery uneconomical and energy-inefficient [14]. Moreover, one of the main drawbacks of this process is the high energy consumption required to reach such elevated temperatures. However, during the last years, some advancements have been made to enhance recycling efficiency and reduce energy consumption [15]. Hydrometallurgical processes enable the recovery of strategic metals in the form of hydroxides, after co-precipitation in alkaline environment, or metal salts through leaching steps employing various organic (such as citric, malic, and oxalic acids) and inorganic (such as hydrochloric, sulfuric, and nitric acids) systems [12]. Compared to pyrometallurgical processes, they offer improved metal recovery efficiency, enhanced selectivity, and reduced energy consumption. In fact, they operate at low temperatures, generally near room temperature [14]. On the other hand, these processes are associated with the generation of contaminated wastewater that must be properly treated, as well as significant acid consumption [11,16].

Looking at the alternative systems other than water for the dissolution of elements and compounds from BMs, the entire scientific community has pointed to the use of Deep Eutectic Solvents (DESs). However, the term DES is often used without proper solid–liquid phase diagrams, and there are no standardized established criteria distinguishing deep from non-deep eutectics, making the classification somewhat subjective. According to Crespo et al. [17], if a eutectic mixture (characterized by a melting point lower than the one of the former constituents) is considered, and this mixture shows an experimental eutectic point lower than the theoretical one, it is possible to address such mixture as a DES. DESs usually show peculiar chemical–physical properties, high viscosity, and enhanced solvent ability. These features are the result of the formation, at molecular level, of an intense network of hydrogen bonds, combined with variable rates of other intermolecular interactions (dipole–dipole, London, Wan der Walls). However, an almost infinite number of mixtures between hydrogen bond donors (HBDs) and acceptors (HBAs) exhibiting low melting points can be prepared, independently to their DES status. To broadly categorize these complex solvents, the term Low Melting Mixtures Solvents (LoMMSs) was recently proposed by Chen and Yu [18]. Thus, considering a general LoMMSs, many systems showing good potential for efficiently leaching metallic species from lithium-ion batteries (LIBs) can be prepared, as confirmed by an exponential surge in research over the past two decades. A pivotal study by Abbott et al. [19] in 2006 was among the first to demonstrate the dissolution of metal oxides using choline chloride-based eutectics. More than a decade later, Tran et al. [20] applied this approach to processing cathode materials. Since then, the application of LoMMSs in spent-LIB recycling has grown exponentially and the number of the corresponding reports consistently increased [21,22,23].

The aim of the present review paper is to provide a useful summary of the recently reported LoMMSs for the extraction of Li, Co, Ni, and Mn by the processing of BM based on two very common cathodic configurations: LiCoO_2_ (LCO) and LiNi_x_Mn_y_Co_1-x-y_O_2_ (NCM). In this context, Section 2.1 is dedicated to a general summary of the research on LoMMSs published since 2020. Table 1 reports a detailed list of these systems divided for application (LCO, NCM, or both). From Section 2.1 onward, the manuscript systematically reviews the recent literature on the use of LoMMSs for extracting critical metals from lithium-ion battery black mass, with an emphasis on lithium cobalt oxide (LCO) and nickel–cobalt–manganese (NCM) cathodes. Section 2.1 presents a comprehensive table summarizing 71 published studies from 2020 to 2025, categorized by solvent system, temperature, leaching time, and extraction efficiency. In Section 2.1 a short introduction to BM complexity is provided, while Section 2.2 presents a summary of the systems discussed in the present review. Then, in Section 2.3 and Section 2.4 some key categories of LoMMSs, including binary and ternary DESs based on choline chloride, as well as systems that employ alternative hydrogen bond acceptors are discussed in terms of how solvent composition, including the nature of the hydrogen bond donor and acceptor, influences leaching performance, redox behavior, and metal selectivity. Section 2.5 provides a comparative synthesis across all systems, highlighting performance trends in reaction kinetics, selectivity, recyclability, and solvent degradation, while also identifying emerging challenges such as lithium recovery and environmental sustainability.

Finally, the manuscript concludes with future outlooks (Section 3), identifying current gaps and suggesting directions for improving scalability, selectivity, and environmental compatibility in solvometallurgical processes.

## 2. Result and Discussion

### 2.1. Black Mass Composition and Complexity

The composition of BM, the fine powder obtained from the mechanical and thermal processing of spent LIBs, varies significantly depending on the battery’s cathode chemistry, manufacturer, and usage history. For instance, LIBs utilizing lithium cobalt oxide (LCO) cathodes, commonly found in portable electronics, typically yield BM with a higher cobalt content and minimal nickel and manganese. In contrast, batteries with nickel–cobalt–manganese (NCM) cathodes, prevalent in electric vehicles, produce BM with varying ratios of nickel, cobalt, and manganese, depending on the specific NCM formulation (e.g., NCM111, NCM622, NCM811). Additionally, BM often contains substantial amounts of graphite from the anode, as well as other components such as aluminum, copper, and electrolyte residues. These compositional differences influence the efficiency and selectivity of metal recovery processes, including solvometallurgical methods. Table 1 summarizes typical elemental compositions of BM derived from various cathode chemistries, highlighting the inherent variability that must be considered when designing and optimizing recycling strategies [24].

### 2.2. Summary of Published Research

The extraction of Li, Co, Ni, and Mn from BM composed from LCO and NCM cathodes using LoMMSs remains an active research area, with no viable industrial technology developed yet. However, numerous proof-of-concept studies have investigated key factors such as leaching mechanisms, selectivity, optimal recovery conditions, and the role of additives in enhancing extraction. To provide a structured approach to this complex topic, the literature published since 2020 has been reviewed and categorized based on the type of battery material studied (Table 2). When different conditions are discussed in a paper, the best one, allowing for the best extraction efficiencies, was reported (higher extraction yield).

Table 2 provides a summary of key research studies published between January 2020 and February 2025 that explore low melting mixtures used in the solvometallurgical treatment of LCO and NCM BM. The cited papers were identified through a cross-search of the Scopus and Google Scholar databases. In the following sections, the leaching systems reported in Table 1 will be discussed, with a particular focus on any possible mechanistic aspects and recyclability.

### 2.3. Choline Chloride–Based Binary LoMMSs

#### 2.3.1. ChCl:Urea

Among the earliest and most extensively studied deep eutectic solvents for lithium-ion battery recycling, the binary mixture of choline chloride and urea (ChCl:U) has demonstrated notable performance in both leaching efficiency and mechanistic sophistication [28]. Urea, as a hydrogen bond donor, not only contributes to the formation of a stable eutectic mixture but also plays a crucial chemical role as a mild reducing agent, facilitating the conversion of insoluble Co(III) species to the more soluble Co(II) form. This redox behavior significantly enhances the leaching performance of the system.

Experimental studies have shown that the ChCl:U system can achieve lithium and cobalt extraction efficiencies approaching those of conventional hydrometallurgical processes. Specifically, when operated at 180 °C for 12 h, extraction efficiencies reached 94.7% for lithium and 97.9% for cobalt. This performance is particularly noteworthy given that similar efficiencies with ChCl:ethylene glycol (ChCl:EG) require harsher conditions, including temperatures up to 220 °C and reaction durations of 24 h. The superior reducing power of ChCl:U, as confirmed by cyclic voltammetry and supported by Fukui function calculations, explains its enhanced efficiency under milder conditions. The reduction potential of the ChCl:U system ranges from −0.45 to −0.35 V versus Ag, which is significantly more negative than the 0.40–0.50 V observed for ChCl:EG. This stronger reducibility facilitates more effective electron transfer and accelerates the leaching process.

Kinetic analysis further reveals that the leaching process with ChCl:U is governed by solution and electron diffusion rather than surface chemical reaction, which is typical in mineral acid-based systems. Fitting the experimental data to a shrinking core model confirms that diffusion through the DES is the rate-limiting step, particularly under higher solid-to-liquid ratios or lower temperatures. Activation energy calculations yielded values of 120.0 and 123.1 kJ mol^−1^ for lithium and cobalt, respectively, which are higher than those associated with acid or ammoniacal systems. This suggests that while ChCl:U systems require thermal input to reach high extraction efficiencies, their overall environmental and operational profile remains favorable due to the avoidance of harsh chemicals and secondary waste.

A spectroscopic analysis of the leaching process offers insights into the molecular interactions driving metal solubilization. UV-VIS spectra show a characteristic absorption band at 628 nm, consistent with octahedral Co^2+^ complexes such as Co(urea)_2_Cl_2_. FT-IR measurements support this coordination environment, revealing significant shifts in N-H and C=O stretching vibrations upon complexation, as well as new bands associated with Co-O and Co-N bonding. These findings affirm that urea acts as a bidentate ligand, coordinating through both its oxygen and nitrogen atoms to stabilize the dissolved cobalt species.

Following leaching, cobalt can be efficiently recovered from the DES by a dilution–precipitation–calcination process. Using precipitants such as oxalic acid or sodium hydroxide, the cobalt is converted into crystalline Co_3_O_4_ with particle sizes below 300 nm. X-ray diffraction confirms the formation of cubic spinel structures, while XPS analysis verifies the oxidation state and chemical integrity of the recovered material. Notably, precipitation with sodium carbonate resulted in lower crystallinity and retained sodium contamination, indicating oxalic acid and sodium hydroxide as more suitable recovery agents. The ChCl:U system offers a compelling balance of chemical effectiveness and environmental compatibility. Its strong reducing power, coupled with favorable coordination chemistry and straightforward post-leaching recovery, positions it as a promising candidate for scalable and sustainable solvometallurgical processing of spent lithium-ion batteries.

#### 2.3.2. ChCl:Polyol Systems

Choline chloride (ChCl) combined with polyols such as ethylene glycol (EG) or glycerol forms some of the most widely investigated DESs for the solvometallurgical recovery of metals from lithium-ion battery BM. These binary systems, often typified by the ChCl:EG eutectic known as “ethaline”, have been shown to dissolve transition metal oxides with notable efficiency, though their performance is highly dependent on the processing conditions and solvent integrity. Early studies established that the ChCl:EG system achieves high extraction efficiencies only at elevated temperatures and prolonged durations. For instance, at 220 °C for 24 h, Co extraction from LCO reached 94.1% and Li extraction approached 89.8% [27]. However, under milder conditions, efficiencies drop sharply, with less than 30% Co recovered at 160 °C over the same period, suggesting a strong dependence on thermal activation to facilitate metal dissolution.

Later investigations provided key insights into the chemical dynamics of this system. Spectroscopic analysis demonstrated the formation of [CoCl_4_]^2−^ complexes, evidenced by characteristic UV-Vis absorption bands between 631 and 690 nm, indicating that the dissolution mechanism involves complexation between metal cations and chloride anions from ChCl [53]. Notably, EG itself has minimal proton activity, and thus contributes primarily through weak coordination rather than acid-driven solubilization [78]. A major breakthrough in understanding came with the realization that thermal decomposition of the DES plays a decisive role in improving extraction performance. As shown by Schiavi et al. [79], thermal treatment at temperatures above 180 °C leads to the in situ generation of trichloride ions (Cl_3_^−^)—species that exhibit oxidative properties capable of enhancing the dissolution of Co, Ni, and Mn oxides. These decomposition products were not merely detrimental artifacts but served as active agents in an oxidative leaching mechanism, akin to that observed in halogen chemistry. Despite these advantages, the same decomposition process poses a challenge to reusability. Extended use or repeated heating leads to the accumulation of toxic byproducts such as 2-methoxyethanol and chlorinated ethers, which compromise both the green credentials and the structural integrity of the DES. Furthermore, with repeated cycling, the selectivity of metal extraction degrades. Initially, the ChCl:EG system favors Co and Mn over Ni; however, after reuse, Ni extraction increases significantly, complicating downstream separation [78]. Efforts to mitigate this issue have included strategies such as shorter leaching durations and the design of modified DES systems pre-loaded with Cl_3_^−^ anions through electrochemical activation, thereby avoiding thermal decomposition altogether. These innovations represent promising avenues for improving DES stability while retaining high metal extraction performance. Additional studies employing alternative polyols such as glycerol (GLY) and polyethylene glycols (PEGs) in combination with choline chloride have corroborated the general performance trends observed with ChCl:EG systems. For instance, Yu et al. [28] demonstrated that a ChCl:GLY mixture achieved Co extraction efficiencies of up to 96% when operated at 200 °C for 20 h. However, these systems often require relatively harsher thermal conditions to achieve efficiencies comparable to aqueous mineral acids, highlighting a trade-off between environmental benefit and energy input. Similarly, DESs based on ChCl and PEG200 have shown effective cobalt extraction capacities (up to 96.9% Co at 80 °C for 24 h), although the viscosity and limited acidity of PEG-based systems tend to slow dissolution kinetics [27]. These findings suggest that while ChCl:polyol systems generally possess lower volatility, improved thermal stability, and excellent metal ion complexation potential (advantageous for large-scale applications), they are also constrained by the need for high processing temperatures and often slower leaching rates. Moreover, PEG-based DESs, while effective, present challenges related to recyclability due to PEG’s molecular weight and difficulty in regeneration from metal-laden solutions [27,28]. Despite these limitations, the simplicity of synthesis, low toxicity, and biodegradability of ChCl:polyol DESs continue to make them attractive candidates for green metallurgical processes. Their tunable properties allow customization for target metal selectivity, and emerging strategies, such as pre-conditioning DESs with oxidants or combining with co-solvents, which may significantly broaden their operational windows. ChCl:polyol binary systems, particularly ChCl:EG, offer a compelling combination of high metal extraction efficiency, environmental sustainability, and process adaptability. Their effectiveness is notably enhanced through mechanisms such as chloride coordination, thermal activation, and in situ formation of oxidative species like Cl_3_^−^, though this advantage is balanced by challenges in long-term solvent reusability and chemical stability. As evidenced across multiple studies further research into mechanistic understanding, degradation control, and solvent regeneration techniques is crucial to advance these systems from laboratory viability to industrial adoption in sustainable lithium-ion battery recycling [53,78].

#### 2.3.3. ChCl:Organic Acids

Deep eutectic solvents composed of ChCl as HBA and organic acids as HBDs represent a promising class of green lixiviants for the selective and efficient recovery of valuable metals from spent lithium-ion batteries. These systems exploit both the coordinating ability of chloride ions and the acidity and redox activity of organic acids to break down complex oxide structures, dissolve metal cations, and facilitate their separation and reuse. The coordination and acid-base properties of organic acids critically determine the efficiency and selectivity of these systems. For instance, citric acid-based DESs (ChCl:citric acid) have shown excellent leaching capabilities under hydrated conditions, achieving up to 97.6% Co and 100% Li extraction from LiCoO_2_ (LCO) cathodes. The presence of water not only lowers viscosity and improves solute transport but also modulates the coordination environment, enabling better solubilization and subsequent metal recovery. Furthermore, these hydrated DESs demonstrated recyclability, with recovered solvents maintaining metal leaching performance over multiple cycles and enabling efficient cobalt precipitation through speciation control [56]. Similarly, systems incorporating oxalic acid (ChCl:oxalic acid) have been extensively studied due to oxalic acid’s strong chelating and mild reducing properties. These systems operate effectively even at moderate temperatures (60–90 °C), providing over 99% Li and 94% Co leaching from both LCO and NCM cathodes within short durations (15–30 min) [59,60]. Oxalate complex formation is central to the dissolution mechanism, as evidenced by FTIR and XPS analyses showing coordinated metal-oxalate species in solution. The chelation not only aids in dissolution but also enables selective recovery strategies: Ni precipitates as readily as oxalate while Co and Mn remain solvated, allowing for staged separation The selectivity of these systems can be further tuned through ligand environment engineering. Chang et al. [59] demonstrated that by adjusting the coordination chemistry of ChCl-oxalic acid systems, Co, Ni, and Mn could be differentially solubilized and recovered in a stepwise fashion. Nickel precipitated as NiC_2_O_4_·2H_2_O, while Co and Mn were retained in solution, and subsequently recovered with high purity as Co_3_O_4_ and Mn_3_O_4_, respectively. These findings underscore the importance of ligand–metal interactions and suggest that organic acid-based DESs are not merely leachants but active participants in coordination-driven separations. Beyond carboxylic acids, sulfonic and phosphinic acid systems (e.g., ChCl:p-toluenesulfonic acid and ChCl:phenylphosphinic acid) offer enhanced leaching efficiency due to their stronger acidity and unique binding capabilities. Ebrahimi et al. [66] reported that a ternary system of ChCl, ethylene glycol, and p-toluenesulfonic acid (PTSA) achieved high metal recoveries from NCM materials (97% Co, 96.4% Ni, 93% Mn) under mild conditions (100 °C, 72 h), driven by the high current density and redox activity of PTSA, confirmed by cyclic voltammetry. Similarly, phenylphosphinic acid-containing DESs were shown to form stable metal–ligand complexes involving P=O coordination, enabling efficient leaching of transition metals and distinguishing Co and Ni coordination geometries via spectroscopic methods [56]. The ChCl:L-ascorbic acid system adds a redox dimension to the process. Hua et al. [58] used ionization potential calculations and cyclic voltammetry to design DESs with high reducing power, confirming that L-ascorbic acid, with a lower ionization potential, efficiently reduces and leaches Co, Ni, Mn, and Li under mild conditions (50 °C, 1 h). This not only exemplifies a rational solvent design approach but also affirms the importance of pairing ChCl with chemically compatible and redox-active donors. In terms of reusability and process integration, several studies illustrate the regeneration of both solvent and metal precursors directly within the DES matrix. Luo et al. [60,61] demonstrated a double closed-loop process using ChCl:oxalic acid, where DES was reused multiple times without performance loss, and high-purity NCM precursors were regenerated in situ from leachates. Electrochemical performance testing of the regenerated materials confirmed parity with commercial standards. Finally, the selectivity of ChCl-based DESs for cobalt over other metals such as nickel or iron has been utilized for targeted cobalt recovery. Schiavi et al. [78] employed a ChCl:EG system for selective Co leaching from mixed LIB materials, achieving 90% Co and only 10% Ni dissolution, and demonstrated solvent reuse with consistent performance across cycles.

#### 2.3.4. Ternary Systems Based on ChCl

Ternary ChCl-based deep eutectic solvents (DESs) represent a strategic advancement in the field of lithium-ion battery (LIB) recycling. These systems combine ChCl with two hydrogen bond donors or one donor and an additive (often an oxidant or modulator), achieving superior control over leaching efficiency, redox behavior, and selectivity compared to binary systems. By fine-tuning the solvent environment, ternary DESs simultaneously enhance dissolution rates and support recovery and regeneration processes, making them highly suitable for industrial applications.

One of the most extensively investigated ternary systems is ChCl:ethylene glycol:acid (e.g., maleic, citric, tartaric, or p-toluenesulfonic acid). These systems exploit the synergetic effects of chloride complexation, hydrogen bonding, and acidity to solubilize metal oxides under relatively mild conditions. For example, Li et al. reported that a ChCl:maleic acid:EG system achieved over 98% recovery for both Co and Li from LCO at 150 °C within 10 h, and retained over 90% of its leaching efficiency even after four reuse cycles, highlighting its recyclability and high metal loading capacity (up to 59 g/L) [25]. Microwave-assisted DESs have also gained attention for their rapid energy delivery and enhanced leaching kinetics. Ma et al. designed a ChCl:oxalic acid:water system that achieved over 99% Li extraction and 99.2% Co recovery from LCO within just 10 min at 100 °C. Mechanistic investigation revealed the formation of a transient Co-polymer complex that hydrolyzes upon water dilution, facilitating in situ precipitation of high-purity cobalt oxalate without external precipitants. The system was reusable over four cycles without significant loss in efficiency [30]. The inclusion of strong sulfonic acids such as p-toluenesulfonic acid (PTSA) in ChCl-based ternary mixtures also yields highly effective solvents. Roldán-Ruiz et al. demonstrated that a ChCl:PTSA:water system could dissolve over 94% of Co from spent LIBs at 90 °C within 15 min, and allowed for the subsequent precipitation and calcination of Co_3_O_4_ from the leachate. The DES was low-viscosity and operated without the need for additional reductants, making it appealing for industrial scalability [33]. In a related study, Liao et al. formulated a novel ChCl:benzenesulfonic acid:ethanol ternary DES, which achieved Li and Co leaching efficiencies of 99% and 98%, respectively, under mild conditions (90 °C, 2 h). Kinetic modeling confirmed that the leaching process was surface-reaction-controlled, and cobalt was recovered as Co_3_O_4_ via precipitation with oxalic acid and NaOH [39]. The mechanistic depth of ternary systems is further exemplified in the ChCl:EG:tartaric acid system, which was engineered by Cheng et al. [67] to enable sequential metal separation. This system, when integrated with electrodeposition, allowed for the orderly recovery of Li^+^, Ni^2+^, and Co^2+^ as Li_2_CO_3_, NiO, and Co(OH)_2_, respectively. This multi-functional solvent served not only as a leachant but also as a coordinating and selective separation medium, and was regenerated post-electrodeposition for reuse. Meanwhile, Dong et al. [69] presented a ChCl:malonic acid:EG ternary DES with low viscosity and strong reducing capability, achieving 100% extraction efficiency for Li, Ni, Co, and Mn. The system facilitated co-precipitation of transition metals and subsequent re-synthesis of cathode materials, demonstrating a direct link between leaching chemistry and cathode regeneration. Lastly, efforts to enhance selectivity using oxidative additives and ionic liquids were explored by Domańska et al. [68] who incorporated glycine and hydrogen peroxide into ChCl:malonic acid systems. This approach enabled staged extraction of Co, Ni, Mn, and Li with efficiencies reaching 100% in optimized multi-step protocols. These systems also show promise in coupling DESs with ionic liquids for hybrid extraction technologies.

### 2.4. Systems with Alternative Hydrogen Bond Acceptors than ChCl

The diversification of deep eutectic solvents (DESs) by employing hydrogen bond acceptors (HBAs) beyond choline chloride (ChCl) represents a strategic evolution in solvometallurgical processes for LIB recycling. These systems leverage alternative HBAs—such as quaternary ammonium salts (e.g., TBAC, TEAC), amides (e.g., urea analogs), or sulfonium derivatives—offering new physicochemical environments that can tune solubility, redox potential, and coordination dynamics of target metals.

#### 2.4.1. Mechanistic Insights and Coordination Chemistry

The fundamental dissolution mechanism in DES-mediated leaching involves several interconnected steps: protonation of surface oxides, reduction in multivalent metal species, and stabilization of the resulting cations via complexation. In systems employing HBAs such as TBAC (tetrabutylammonium chloride) or TEAC (triethylammonium chloride), the larger steric bulk and hydrophobic alkyl chains create a less polar environment. This can reduce hydrogen bonding density in the bulk phase and facilitate greater availability of proton donors and chloride ions at the solid–liquid interface, thereby promoting more efficient oxide protonation and ion solubilization [32,56]. Kinetic modeling, often fitted to the Jander diffusion model, demonstrates that these systems exhibit diffusion-controlled behavior, consistent with the DESs where solvent structuring around solid particles governs reaction rates more than intrinsic redox kinetics. For instance, in the TBAC:MCA system, high recovery rates were achieved at 100 °C over several hours, yet activation energy data suggests significant mass transfer resistance in the absence of water or co-solvents [84]. The coordination environment plays a pivotal role in the stability of dissolved metal species. For example, acetylsalicylic acid (ASA), used with TEAC, introduces both carboxylic and phenolic functionalities that enhance metal chelation. The combination of proton-driven oxide dissolution and ligand-assisted stabilization leads to rapid and nearly quantitative recovery of Li, Co, Ni, and Mn within minutes at mild temperatures [56]. These multifunctional donors form stable chelates while simultaneously lowering solution pH, accelerating oxide lattice breakdown.

#### 2.4.2. Eutectic Formation and Its Relationship to Efficiency

Eutectic formation is central to DES performance. The eutectic point, where the DES reaches its minimum melting temperature, often corresponds to the optimal balance between solvation capacity, viscosity, and ionic mobility. Efficient eutectic formation ensures a homogeneous liquid phase with enhanced diffusivity and minimizes internal hydrogen bonding that could otherwise sequester the reactive species. Non-ChCl-based DESs often exhibit broader eutectic ranges due to the flexible steric and electronic configurations of their HBAs. For instance, EG:maleic acid and EG:citric acid systems demonstrate high leaching efficiency at relatively low temperatures, largely because the eutectic phase remains liquid under mild conditions and supports favorable metal–organic complex formation [32]. Moreover, deviations from the eutectic ratio can dramatically alter performance. Above the eutectic threshold, the mixture may phase-separate or crystallize under operating temperatures, reducing effective contact with the cathode surface. Conversely, sub-eutectic compositions may suffer from elevated viscosity and limited mass transport.

#### 2.4.3. Role of Water and Hydration Effects

Water incorporation is another critical factor influencing both leaching kinetics and solvent stability. In trace amounts, water can reduce viscosity, enhance ion mobility, and facilitate the hydrolysis of metal–ligand complexes, especially for transition metals like Co and Mn, which readily form aquo-complexes. This has been demonstrated in systems like TBAC:MCA and TEAC:ASA, where the addition of ≤10 wt.% water significantly improved metal recovery while maintaining the structural integrity of the DES [32,56]. However, excessive water disrupts hydrogen bond networks and may shift the equilibrium away from the eutectic state. In such cases, phase separation or decreased solvation efficiency can occur, leading to inconsistent leaching and lower selectivity. Some studies have also shown that increased water content can reduce the reducing potential of DESs, thereby slowing the dissolution of higher-valence metal species like Co^3+^ or Mn^4+^. A nuanced effect is also observed in the precipitation and recovery steps: aqueous dilution of metal-loaded DESs can promote selective crystallization or co-precipitation of metal salts (e.g., oxalates or carbonates), simplifying downstream separation. This dual utility of water, as a kinetic accelerator and recovery aid, makes it an important variable in process design.

#### 2.4.4. System Stability, Regeneration, and Environmental Considerations

Non-ChCl HBAs often confer enhanced oxidative and thermal stability compared to ChCl-based systems, extending solvent lifespan across multiple cycles. For example, TEAC:ASA retained leaching efficiency across several regeneration cycles without signs of degradation or discoloration, a typical proxy for oxidative breakdown [56]. Moreover, the reduced halide content in some alternative systems (e.g., those based on lactones or sulfonium salts) minimizes the risk of corrosive byproduct formation during operation or disposal. From an environmental perspective, several non-ChCl DESs also utilize bio-based or biodegradable HBAs, aligning well with circular economy goals in battery material recovery. Systems utilizing ethanolamines, betaine derivatives, or sulfur-containing analogs have demonstrated comparable efficiencies to ChCl-based systems, while offering lower aquatic toxicity and easier disposal protocols [36,42].

### 2.5. Comparative Observations and Highlights

A comparative evaluation of the LoMMSs discussed in Section 2.2, Section 2.3 and Section 2.4 reveals consistent trends that underscore the interplay between solvent composition, leaching kinetics, metal selectivity, and process sustainability. These observations illuminate both the performance strengths of current DES formulations and areas where further optimization is needed to support industrial implementation. The highest-efficiency systems for leaching LiCoO_2_ (LCO) cathodes consistently involve ChCl-based DESs combined with strong organic acids. Notably, ChCl:oxalic acid (ChCl:OA) and ChCl:formic acid (ChCl:FA) systems achieved complete leaching of both lithium and cobalt at relatively low temperatures: 100% extraction was reported at 90 °C for 2 h in ChCl:OA, and at just 70 °C over 12 h in ChCl:FA [32,56]. A hydrated ternary variant, ChCl:OA:H_2_O, achieved the same result in only 10 min at 100 °C [42]. These systems benefit from the dual functionality of the organic acid as both a proton source and complexing ligand, enabling one-pot, reductant-free leaching. Temperature and solvent composition play critical roles in extraction kinetics. A clear inverse relationship exists between reaction time and operational temperature: while ChCl:glycerol (ChCl:GLY) required 20 h at 200 °C to approach 96% Co extraction [47], ternary systems such as ChCl:EG:lactic acid or ChCl:EG:tartaric acid achieved nearly complete metal recovery in just 10 min at 120 °C [32,56]. The introduction of water or a third component often reduces viscosity, enhances ionic mobility, and facilitates faster and more homogeneous interaction with the cathode surface. Ternary and hydrated DESs thus consistently outperform binary systems in leaching kinetics under moderate thermal regimes. Solvent composition also governs metal selectivity. For instance, ChCl:EG:urea exhibited preferential lithium extraction (97%) over Co, Ni, and Mn (all below 45%) at 40 °C across 72 h, likely due to limited redox activity and weak transition metal coordination by EG and urea [32,42].

In contrast, strongly acidic or redox-active systems such as ChCl:FA and ChCl:OA promote simultaneous dissolution of Li and Co through electron transfer or ligand-driven lattice destabilization. This pattern highlights that acid strength alone is insufficient to predict performance coordination strength and redox chemistry are equally decisive.

Further comparisons show that DESs with the fastest leaching kinetics include ChCl:OA:H_2_O for 10 min at 100 °C [42], PTSA:ChCl:H_2_O for 15 min at 90 °C [55], and (ChCl:EG:TAR) 10 min at 120 °C [78]. These systems combine proton availability, low viscosity, and strong coordination chemistry, making them strong candidates for time-efficient and scalable applications. Mechanistic investigations further differentiate these systems. Redox-active DESs, particularly those containing formic acid or sulfamic acid (SSA), exhibit “dot-etching” behavior, in which localized reduction of Co^3+^ to Co^2+^ initiates structural disintegration and generates surface porosity. Acid-driven systems such as ChCl:OA are instead characterized by a layer-peeling dissolution, whereby progressive proton attack and complex formation lead to structural collapse. Kinetic modeling—using shrinking-core or interface-controlled models—has validated that cobalt dissolution often follows interface-reaction pathways, while lithium follows diffusion-limited kinetics, especially in systems like EG:SSA·2H_2_O [31]. A critical aspect for practical deployment is solvent reusability. While many LoMMSs demonstrate high initial efficiency, long-term reuse remains variable. Hydrated systems such as ChCl:CIT:H_2_O and ChCl:OA:H_2_O retained extraction capacity over four cycles with minimal degradation. In contrast, thermally sensitive systems like ChCl:EG showed notable declines in efficiency and selectivity after repeated use, attributed to EG oxidation and chloride decomposition at temperatures exceeding 180 °C. Systems that rely heavily on polyols or PEGs are particularly susceptible to thermal degradation and may require regeneration protocols or stabilizing additives. Conversely, DESs operating under lower temperatures with co-solvents like water tend to exhibit greater chemical stability and recyclability.

## 3. Future Outlooks

Although LoMMSs have shown considerable potential for the recovery of strategic metals from lithium-ion battery black mass, several limitations still hinder their transition to large-scale application. A major challenge is solvent recyclability, which remains inconsistent across different systems. In many cases, the strong coordination capacity of hydrogen bond acceptors, particularly those based on polyols, amides, or organic acids, leads to the formation of stable metal–ligand complexes that complicate the separation and isolation of recovered metals. This strong binding can hinder subsequent purification steps, raising concerns over the efficiency and circularity of the process. Furthermore, while some solvent systems, as those involving citric or oxalic acid, demonstrate promising reusability, others, particularly those requiring high operating temperatures, degrade over time or require frequent regeneration, thus affecting long-term sustainability. Another critical gap lies in the ambiguity surrounding the true necessity of deep eutectic solvents (DESs) in many reported systems. Often, it remains unclear whether the enhanced performance stems from eutectic behavior or simply from the chemical reactivity of the components in a non-ideal mixture. This raises fundamental questions about whether a complex eutectic system is needed at all, or if simpler binary or ternary low-melting mixtures could offer similar or better performance with improved recyclability and lower viscosity. Further thermodynamic and structural characterization of these mixtures is needed to distinguish between “true” DESs and effective but non-eutectic formulations. Moreover, selectivity remains a significant hurdle, particularly in the recovery of metals from NCM-type BM, where the co-presence of Li, Ni, Co, and Mn introduces complex redox and coordination interactions. Although some solvents show preferential leaching behavior, there is a clear need for systems that can achieve fine-tuned, metal-specific selectivity to reduce downstream separation steps. Process conditions also require optimization: many promising systems still rely on long extraction times or elevated temperatures, which could pose scalability and cost-efficiency issues. Finally, a deeper mechanistic understanding of how solvent composition influences leaching kinetics and speciation, particularly for multivalent metals, is essential. Linking solvent design to targeted metal coordination environments could enable the rational development of next-generation LoMMSs that are not only effective but also more environmentally benign and compatible with closed-loop recovery systems. Addressing these challenges will be pivotal in advancing solvometallurgy from laboratory-scale innovation to a core component of future battery recycling infrastructure.

While solvometallurgy offers a promising alternative to traditional pyrometallurgical and hydrometallurgical approaches, its future development must also account for environmental sustainability across the full process lifecycle. Although DESs are often marketed as “green” solvents due to their low volatility and tunable properties, their chemical composition and degradation products deserve closer scrutiny. Some commonly used components, such as ethylene glycol, urea, and certain sulfonic acids, can generate potentially hazardous byproducts or exhibit aquatic toxicity when improperly managed. Moreover, the energy footprint of solvometallurgical processes is highly dependent on the required leaching temperature and residence time. Systems operating above 150 °C for extended periods (e.g., ChCl:EG or ChCl:GLY) may offset their chemical advantages through increased energy consumption, unless paired with renewable energy sources or heat recovery systems. Thus, prioritizing low-temperature and short-duration DESs, such as those leveraging formic acid or oxalic acid, can help minimize both energy input and greenhouse gas emissions. Operational simplicity and utility demand also influence the practical scalability of these systems. Ternary and hydrated DESs often reduce viscosity and enable milder operating conditions, but their formulation and regeneration may involve additional steps (e.g., solvent purification, solid separation, rebalancing of DES ratios). Processes must be streamlined to ensure industrial viability and minimize reliance on intensive infrastructure. Water and utility consumption further complicate the picture. While many DESs function under anhydrous conditions, water is frequently introduced to modulate viscosity or enable precipitation and purification steps. Therefore, closed-loop water integration and solvent recovery systems are essential to prevent excessive consumption and downstream wastewater burdens. Finally, waste management, including both aqueous waste and solid residues, requires systematic evaluation. DESs degrade over time, and their disposal must be managed to avoid introducing persistent organics into the environment. Strategies such as solvent recycling, metal recovery through selective precipitation or electrodeposition, and valorization of residual solids should be integral to process design. Altogether, the future of solvometallurgy depends not only on metal recovery efficiency but also on its ability to meet environmental and economic sustainability benchmarks. Standardized life-cycle assessments (LCAs), green chemistry metrics, and regulatory frameworks will be key tools to guide the responsible implementation of LoMMSs in LIB recycling.

## Figures and Tables

**Table 1:** Typical elemental composition of black mass from different cathode chemistries [24].

Cathode Type	Li (wt%)	Co (wt%)	Ni (wt%)	Mn (wt%)	Graphite (wt%)	Notes
LCO	3–5	20–30	<1	<1	20–25	Common in portable electronics
NCM111	3–4	15–20	15–20	15–20	15–20	Early-generation EV batteries
NCM622	2.5–3.5	8–12	25–30	5–10	10–20	Mid-generation EV batteries
NCM811	2–3	5–7	30–40	2–5	10–20	High-nickel EV batteries

**Table 2:** Summary of the recent publications on solvometallurgy processing of different BMs.

Entry	Solvent	T (°C)	Time	Efficiency (%)	Reference
LCO cathode					
1	MA:EG	150	10 h	Co 98.3; Li 98.4	[25]
2	ChCl:CIT:H_2_O ChCl:EG ChCl:MAL ChCl:MALO ChCl:OA	60	4 h	Co 99.6 Co 2.1 Co 81.2 Co 24.4 Co 19.6	[26]
3	ChCl:U	180	12 h	Li 95; Co 95	[27]
4	ChCl:GLY	200	20 h	Co 95.4	[28]
5	ChCl:OA	90	2 h	Co 100	[29]
6	ChCl:OA:H_2_O	100	10 min	Li 99; Co 99	[30]
7	ChCl:FA	70	10 min	Li 100; Co 100	[31]
8	ChCl:CIT	40	1 h	Co 98	[26]
9	ChCl:MAL:PTSA	100		Li 98.68; Co 98.71	[32]
10	PTSA:ChCl:H_2_O	90	15 min	Li 100; Co 100	[33]
11	GUA:LACT	50	24 h	Li 94.7; Co 96.9	[34]
12	PEG200:PA	80	24 h	Li 98.7; Co 96.9	[35]
13	BTFA:TOPO	70	180 min	Li 93.0; Co 90.6	[36]
14	ChCl:EG	200	12 h	Li 86; Co 95	[37]
15	ChCl:U:EG	100	72 h	Li 92.83; Co 1.61; Ni 0.72; Mn 0.42	[38]
16	ChCl:BSA:EtOH	90	2 h	Li 99; Co 98	[39]
17	PEG200:TU	160	24 h	Co 60.2	[40]
18	PEG200:PTSA H_2_O	100	24 h	Li 99.4; Co 99.5	[41]
19	ChCl:OA	90	2 h	Li 100; Co 100	[42]
20	ChCl:OA 2H_2_O	120	12 h	Li 96.1; Co 96.3	[43]
21	PEG:PTSA	80	24 h	Li 85.7; Co 70.4	[41]
22	PEG:AA	80	24 h	Li 44.6; Co 55.4	[44]
23	EG:TART	120	12 h	Li 98.34	[45]
24	ChCl:OA	120	12 h	Li 96.1; Co 96.3	[43]
25	EG:SSA 2H_2_O	110	6 h	Li 98.3; Co 93.5	[46]
26	ChCl:LACT	105	5 h	Li 95; Co 95	[47]
27	PTSA:PolyEG	100	24 h	Co 99.5	[44]
28	ChCl:EG	87.5	2 h	Li 100; Co 100	[48]
29	ChCl:THBA	110	12 h	Li 98; Co 98	[49]
30	ChCl:CIT:H_2_O	120	4 h	Li 100; Co 97.6	[50]
31	BHC:LACT	120	2.2 h	Li 99.98; Co 99.86	[51]
32	EG:U	157	4 min	Li 91; Co 94	[52]
33	ChCl:GLY	200	20 h	Co 96	[28]
NCM or LCO + NCM cathodes					
34	ChCl:EG	180	24 h	Li 89.94; Co 100; Ni 99.64; Mn 100	[53]
35	BHC:EG	140	10 min	Li 99.5; Co 0.6; Ni 99.7; 34Mn 93.5	[54]
36	BHC:EG	140	20 min	Li 93.6; Co 93.3; Ni 93.0; Mn 82.4	[55]
37	ChCl:TART	70	12 h	Li 96.0; Co 97.1; Ni 98.0; Mn 96.7	[56]
38	ChCl:PPA	100	80 min	Li 97.7; Co 97.0; Ni 96.4; Mn 93.0	[57]
39	ChCl:AA	50	1 h	Li 96.2; Co 98.1; Ni 98.9; Mn 99.3	[58]
40	ChCl:OA	120		Co 95.5; Ni 99.1; Mn 94.5	[59]
41	ChCl:OA	110	2.5 h	Li 99	[60]
42	DMT:OA	60	15 min	Li 100	[61]
43	EG:SSA 2H_2_O	110	6 h	Li 100; Co 94.8; Ni 99.1; Mn 100	[46]
44	ChCl:LACT:CIT	55	3 h	Co 99	[62]
45	ChCl:LACT:H_2_O_2_	45	6 h	Li 86.7; Co 88.5; Ni 84.5	[63]
46	ChCl:PPA:H_2_O_2_	45	6 h	Li 100; Co 98.7	[63]
47	ChCl:PTSA:H_2_O	90	2 h	Li 97.96; Co 100; Ni 99.46; Mn 100	[64]
48	ChCl:AA	120	12 h	Li 100; Co 100; Ni 100; Mn 99.2	[65]
49	ChCl:EG:PTSA	100	72 h	Li 97; Co 97; Ni 97; Mn 97	[66]
50	ChCl:EG:TAR	120	10 min	Co 100; Ni 98.8; Mn 100	[67]
51	ChCl:SUC:TCCA	60	2 h	Li 73.4; Co 53.5; Ni 44.3; Mn 73.4	[68]
52	ChCl:GUC:TCCA	60	2 h	Li 59.9; Co 51.4; Ni 49.6 Mn 65.1	[68]
53	ChCl:CIT:PHM	60	2 h	Li 87.7 Mn 73.7	[68]
54	ChCl:CIT:H_2_O_2_	60	2 h	Li 50.8; Co 51.5 Ni 59.3 Mn 61.8	[68]
55	EG:MA	90	6 h	Li 95.6; Ni 84.6; Co 92.7; Mn 91.3	[69]
56	EG:CIT	95	10 h	Li 99.1 Co 96.2 Ni 97.6 Mn 98.3	[70]
57	EG:DMPT	100	1 h	Li 99.6; Ni 99.3; Co 99.0; Mn 99.4	[71]
58	BHC:CIT	80	0.5 h	Li 99.8; Co 99.8; Ni 99.1; Mn 99.2	[72]
59	BHC:FA	140	6 h	Li 98.0; Co 94.2; Ni 96.0; Mn 92.3	[73]
60	TEAC:ASA	80	2 min	Li 99.1; Co 99.4; Ni 99.6 Mn 99.3	[74]
61	TBAC:MCA	100	7 h	Li 100; Co 100 Ni 100 Mn 100	[75]
62	ChCl:GLU	100	24 h	Li 93.7 Co 94.2 Mn 97.6 Ni 82.4	[76]
63	ChCl:OA	80	2 h	Co 100; Mn 100	[77]
64	ChCl:EG	180	24h	Co 90; Ni 10	[78,79]
65	GH:FUMA	140	40 min	Li 99.8; Ni 98.9; Co 99.5; Mn 96.1	[80]
66	CIT:EG	95	10 h	Li 99.1; Co 96.2; Ni 97.6; Mn 98.3	[81]
67	ChCl:EG:U	40	72h	Li 97; Co 41; Ni 40; Mn 34	[82]
68	ChCl:FA	70	12 h	Li 100; Co 100; Mn 100	[83]
69	ChCl:EG	135	24 h	Li 90; Co 94	[20]
70	EG:OA 2H_2_O	90	12 h	Li 94.4	[84]
71	ChCl:MALE	80	2 h	Li 99.2; Co 65.4; Ni 95.0; Mn 67.9	[85]

ASA: ascorbic acid; BHC: betaine hydrochloride; BSA: benzenesulfonic acid; BTFA: Benzoyltrifluoroacetone; ChCl: choline chloride; CIT: citric acid; DMPT: dimethyl-beta-propiothetin; DMT: dimethyltethyn; EG: ethylene glycol; EtOH: ethanol; FA: formic acid; FUMA: fumaric acid; GH: guanidine hydrochloride; GLU: glucose; LA: levulunic acid; LACT: lactic acid; MA: malic acid; MAL: malonic acid; MALE: maleic acid; MEN: menthol; OA: oxalic acid; PA: Phytic acid; PPA: phenylpropanoic acid; PTSA: paratoluensulfonic acid; SSA: Sulfosalicylic acid; SUC: succinic acid; TCCA: trichloroisocyanuric acid; TEAC: tetraethylammonium chloride; TBAC: tetrabuthylammonium chloride; TAR: tartaric acid; THBA: 3,4,5-Trihydroxybenzoic acid; TOPO: trioctylphosphine oxide; TU: thiourea; U: urea.

## Data Availability

Not applicable.
